# Preparation and characterization of immunopeptides isolated from pig spleen and evaluation of their immunomodulatory properties *in vitro* and *in vivo*

**DOI:** 10.3389/fimmu.2025.1544299

**Published:** 2025-03-18

**Authors:** Minhao Xia, Chong Ling, Hui Ye, Shujie Liang, Qingyun Cao, Weiwei Wang, Changming Zhang, Zemin Dong, Min Tian, Jianjun Zuo, Yongwen Zhu

**Affiliations:** ^1^ College of Animal Science, South China Agricultural University, Guangzhou, Guangdong, China; ^2^ Modern Agricultural College, Xiangxi National Vocational and Technical College, Jishou, China

**Keywords:** pig spleen protein, immunopeptides, enzymatic hydrolysis, immunosuppression, bioactive peptides

## Abstract

The importance of small bioactive peptides derived from pig spleen have been used to enhance immune responses and support intestinal health. However, there is a lack of information regarding the conformational relationship and their effects on immune function of pig spleen proteins (PSPs). The objective of this study was to prepare and assess the immunomodulatory characteristics of immunopeptides from PSP. Firstly, enzymatic hydrolysates from PSP were prepared using alkaline protease and aminopeptidase, and small hydrolysate fractions with a <3 kDa were separated by SDS-PAGE and GPC. The bioactive peptides were then identified at peaks 5 to 7 (PSP-5, 6 and 7) by HPLC and TOF-MS, which were mainly composed of Pro-Glu-Leu by LC-MS. The PSP-5 and PSP-6 pronounced greater beneficial effects on cell viability and nitric oxide (NO) production than PSP-7 in macrophage, and PSP-5 exhibited a higher immunomodulatory ability than PSP-6. *In vivo*, the oral administration of 25-50 mg PSP-5/kg body weight (BW) protected against cyclophosphamide (CTX)-induced immunosuppression in spleen and intestine of mouse, as evidenced by increased cytokine and sIgA productions. In conclusion, a novel set of bioactive immunopeptides derived from PSP through enzymatic hydrolysis could enhance immunomodulatory properties.

## Graphical Abstract

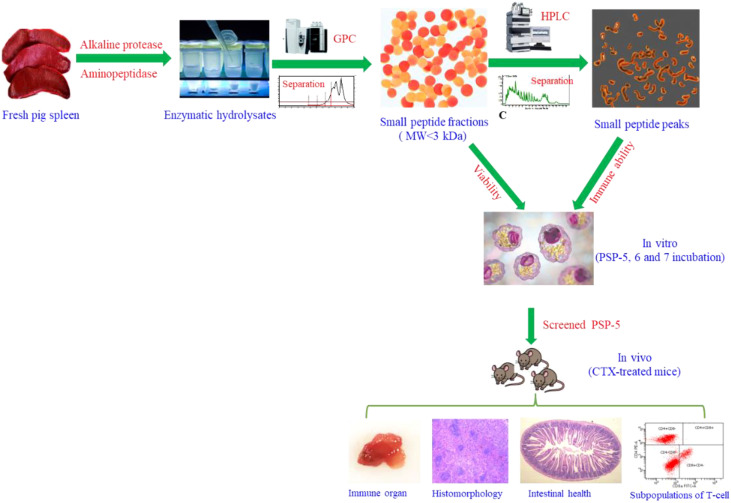

## 1 Introduction

Over the past twenty years, the significance of small peptides has garnered increasing recognition in health and immunity applications (1). The accumulating evidences have documented that immunopeptides enhance the immune function and promote the antibacterial and anti-inflammatory properties in both humans and animals (2). Furthermore, immunopeptides are associated with minimal side effects and low toxicity, owing to their high affinity and specificity for targets, short half-life, and reduced immunogenicity (3). Given these advantages, interest in the application of immunopeptides is rising, whether as pharmaceutical preparations in cancer immunotherapy or as dietary supplements in animal nutrition (3). Numerous studies have highlighted that therapeutic immunopeptides have the remarkable antimicrobial and immunomodulatory abilities relevant to disease diagnosis and cancer immunology by targeting regulatory T cells and natural killer cells (4). The efficacy of immunopeptides is closely linked to their molecular weight (MW), as well as the composition and sequences of amino acids derived from various protein sources and preparation methods (5). Additional immunomodulatory strategies have emphasized that manipulating specific sequences and residue positions of peptides can enhance the activity of immune peptides, thereby facilitating the regulation of immune homeostasis in conditions such as autoimmune diseases and inflammatory responses (6).

Among the various methods for preparing immunopeptides, including artificial synthesis, enzymatic hydrolysis, and microbial fermentation, enzymatic hydrolysis has emerged as an efficient technique for producing protein hydrolysates using either digestive enzymes or enzymes extracted from microorganisms and plants (7). Enzymatic hydrolysis facilitates the breakdown of larger protein molecules into smaller immunoreactive peptides, thereby enhancing their absorption by the body and potentially reducing the allergenic properties of certain proteins for sensitive individuals (8). Recently, there has been a growing emphasis on obtaining immunopeptides from natural functional food sources, which are perceived to offer higher safety and minimize adverse reactions in the body (9). The spleen, recognized as the largest immune organ, is considered a valuable source for generating bioactive peptides, including immunomodulatory and antioxidant peptides. These peptides may exhibit specific immunomodulatory effects and reduce inflammation, thereby potentially supporting the immune system and addressing inflammatory conditions (10). It is important to note that pig spleen models not only exhibit numerous anatomical and physiological similarities to the human immune system, but they are also relatively accessible due to the functional development of porcine spleen as a by-product of pig processing. This feature renders pig spleen a practical and economical source of protein for the production of immunopeptides (11). However, there is a lack of information regarding the conformational relationship and their effects on immune function of pig spleen peptides (PSPs), which further limits the application of PSPs (12). Consequently, the first aim of the present study was to obtain and identify the bioactive immunopeptides derived from PSP based on the enzymatic hydrolysis process and then to explore the specific immunomodulatory properties of the immunopeptides using the models of macrophages *in vitro* and mice *in vivo*.

## 2 Materials and methods

### 2.1 Preparation of enzymatic hydrolysis from PSP

Fresh pig spleens were obtained from a slaughterhouse in Guangzhou, China. Spleen samples were collected from castrated boars (Duroc×Landrace×Large white, 175 days old, 120 kg), immediately frozen in liquid nitrogen, homogenized and ground, and then stored in a refrigerator at -80°C.

Enzymatic hydrolysis was performed using aminopeptidase and alkaline protease. The aminopeptidase was sourced from Novozymes (Copenhagen, Denmark), while the alkaline protease was obtained from SunHY Co., Ltd (Wuhan, China). The activity of aminopeptidase was assessed at 1921 U/g using the Biuret method (13), and the activity of alkaline protease was measured at 21087 U/g via the Lowry method (14). The defatted PSP, which contained 33% protein, was prepared following a previously modified protocol (15). Briefly, the defatted spleen protein sample was combined with a 50 mmol/L Na2HPO4-NaH2PO4 buffer to achieve a pH of 6.5. Subsequently, 900 U/mL of aminopeptidase was added to conduct the enzymatic reaction at 50°C for 4 h, and the mixture was extracted using a subcritical water extractor (Ruishen Instrument Co., Ltd, Shanghai, China). The hydrolysis was conducted under specified conditions, employing an enzyme/substrate ratio of 10056 U/mL of alkaline protease, maintained at a temperature of 48°C and pH 10.0 for a duration of 4 h. The enzymatic reaction was then halted by immersing the mixture in boiling water for 10 minutes. The solution was concentrated to remove insoluble materials and residual enzymes, and subsequently was separated by SDS-PAGE. Finally, the obtained hydrolysates were subjected to fractionation using ultrafiltration membranes (3 kDa). The pig spleen hydrolysate fractions (PSHF), categorized by MW as those below and above 3 kDa, were concentrated, lyophilized, and preserved at-20°C for future *in vitro* studies (Exp. 1).

### 2.2 MW analysis of peptides by GPC

The MW distribution for the various fractions was examined using a TSK gel G2000 SWXL column (300 mm×7.8 mm). A mobile phase composed of a 1:1 blend of acetonitrile (containing 0.1% trifluoroacetic acid) and water (also with 0.1% trifluoroacetic acid) was utilized. The flow rate was kept constant at 0.5 mL/min, and the temperature of the column was adjusted to 27°C. A sample volume of 20 µL was used, with a concentration of 10 mg/mL for the sample.

### 2.3 Separation of peptides by HPLC

Following the process of obtaining hydrolysates with MW less than 3 kDa via the 3 kDa ultrafiltration membrane, a freeze-dried hydrolysate (MW < 3 kDa, 250 mg) was combined with 1 mL of methylene chloride and shaken vigorously. Subsequently, centrifugation was performed at 25,000 × g for 30 minutes. The methanol-water fraction (1 mL) was then concentrated using a Speed Vac (Savant Instruments, NY, USA) for 3 h and later dissolved in 50 mL of distilled water. Portions of 25 mL from the water-soluble fractions were injected into a Delta Pak C18 column (100 Å, 30 × 150 mm) with the Waters HPLC system, as previously detailed by Cao et al. (16). A gradient elution was executed using a mixture of solvent A (0.1% trifluoroacetic acid (TFA), v/v) in deionized water and solvent B (0.1% TFA in acetonitrile, v/v). The elution of peptides occurred as follows: from 0-80 minutes, 40% B; from 80-85 minutes, 40-100% B; from 85-90 minutes, 100% B; and from 90-100 minutes, 100-0% B, with the HPLC system equilibrated for 10 minutes using 100% A. Records and calculations of the proportions of separated fractions from main peaks numbered from 1 to 10 were conducted. Subsequently, these peak fraction samples were collected, lyophilized, and preserved at -20°C for *in vitro* cell culture (Exp. 2).

### 2.4 Component analysis by UPLC/Q-TOF-MS

To investigate the compositional variations among the different PSPs, we utilized an Acquity UPLC HSS T3 column (100 mm × 2.1 mm, 1.8 μm) for sample analysis. The mobile phase consisted of a 0.1% formic acid solution in acetonitrile. The column’s temperature was maintained at 40°C, and a 2 μL injection volume was used. The elution flow rate was fixed at 0.3 mL/min. Electrospray MS detection was performed in both positive and negative ion modes, utilizing voltages of 5.0 kV and 4.0 kV, respectively. During each data acquisition cycle, the most intense molecular ions with intensities greater than 100 were chosen for obtaining the corresponding secondary mass spectrometry data. The acquisition started with a range of m/z 50 to 1200, using a collision energy of 30 eV, and was set to collect 10 secondary spectra every 50 ms.

### 2.5 Identification of peptides by LC-MS

To determine the amino acid sequences of the isolated bioactive peptides, the selected peaks underwent collection and purification via HPLC on the same column, repeated three times with the identical gradient. The samples that were collected were subsequently analyzed for their molecular masses using Ion Trap-Mass Spectrometry (Thermo, MA, USA) and the amino acid sequences were assessed with an automatic protein sequencer (PPSQ-53A), as previously outlined (17). The samples were solubilized in 10% acetic acid and introduced through a stainless steel capillary (100 mm i.d.). To aid the formation of submicron droplets, a stream of air (pneumatic nebulization) was applied. These droplets were vaporized at the interface with nitrogen gas, resulting in the generation of highly charged ions detected by the analyzer. Mass charges were utilized across the entire range of the instrument (0 to 2470 amu). Basic algorithms correlated the charges generated by the compounds to their respective molecular masses. Following the determination of the molecular mass for various fragments, the amino acid sequences of the immunopeptides were acquired within the m/z range of 50-1500 and identified using the SEQUEST sequencing algorithm.

### 2.6 Cell culture, viability assay and immunomodulatory properties

To assess the immunomodulatory influence of pig spleen hydrolysates (PSH; Exp. 1) and the different types of peak fractions (PSP; Exp. 2), totally three MW groups (control, MW < 3 kDa, MW > 3 kDa) as well as eleven PSP groups (control and PSP 1-10 groups). were chosen to evaluate the cell viability. Three independent experiments were performed including four replicates per group in each experiment. RAW 264.7 macrophages were cultured in a basal medium consisting of DMEM, 4 mM L-glutamine, 1 mM sodium pyruvate, and 10% (v/v) FBS (Gibco, NY, USA) for 12 h prior to treatment. The cells were subsequently exposed to various sizes of PSH groups and multiple types of PSP groups. A group that did not undergo peptide incubation served as the control. The cell culture was maintained in a controlled environment with 5% CO_2_ at 37°C for a period of 24 h. Then, the cell viability was evaluated using the CCK-8 assay (MIKX, Shenzhen, China) to identify the effect of different PSP sources incubation. Subsequently, the levels of nitric oxide (NO) and the expression of mRNA for cytokines were further examined to demonstrate the immunomodulatory effects.

### 2.7 Animal immunological responses

All animal handling procedures adhered strictly to the protocol established by the Animal Management Committee of Guangdong Province and South China Agricultural University, China (SCXK 2017-0002). In Exp.3, a total of forty male Balb/c mice were randomly allocated into five groups with comparable body weights (BWs) in each group (23.5 ± 0.5 g; 8 mice per group and 2 mice each replicate cage): the control group (Control), and four groups receiving intraperitoneal injections of cyclophosphamide (CTX) at varying doses: 12.5 mg/kg BW (CTX + PSP-5 12.5), 25 mg/kg BW (CTX + PSP-5 25), and 50 mg/kg BW (CTX + PSP-5 50). Thirty-two mice underwent immune suppression induction through an intraperitoneal injection of CTX at a dosage of 100 mg/kg BW, as previously described (18). Following this, the PSP-5 fraction was administered orally at different dosages of 0, 12.5, 25, or 50 mg/kg BW. Eight additional mice received saline solution to serve as the control group. All animals had unrestricted access to standard food pellets and tap water at room temperature for 4 h. Daily recordings were made of body weight, food intake, and mortality. After 2 weeks post-treatment, one mouse from each replicate cage was euthanized, and samples of thymus and spleen were collected for assessing cellular immune responses. Portions of spleen, jejunum and cecum (the same sections from each mouse) were preserved in 10% neutral buffered formalin, while the mucosal membrane of the jejunum was scraped, quickly frozen in liquid nitrogen, and subsequently stored at-80°C.

### 2.8 Histologic evaluation

Following a 24 h fixation period, samples of the spleen, jejunum, and cecum underwent dehydration, were embedded in paraffin, sectioned, and stained with the conventional hematoxylin-eosin (HE) solution. The sections were examined and captured for histopathological analysis using a microscope (Eclipse E100 and DS-U3, Nikon, Tokyo, Japan).

### 2.9 Assays of the contents of NO and cytokines

The frozen cell and tissue samples were thawed, weighed, and homogenized (Kinematica AG, Switzerland) in sterile physiological saline (1:6, w/v) while kept on ice. Following this, the mixture was centrifuged at 2,000×g for 40 minutes at 4°C. The resulting supernatant underwent an additional centrifugation at 2,000×g for 20 minutes at 4°C to collect for cytokine analysis. NO levels were assessed using fluorescent probes in cells, as previously described (19). The concentrations of TNF-α, IL-1α, IL-2, IL-6, IL-12, and sIgA were measured utilizing the Enzyme-Linked Immunosorbent Assay (mlbio Co., Ltd, Shanghai, China) in accordance with the manufacturer’s guidelines. All measurements were reported as per milligram of protein, which was assessed using the Bradford method.

### 2.10 Flow cytometry analysis

Flow cytometry analysis was conducted using a FACS Aria II flow cytometer (BD Biosciences, San Diego, CA), which features two lasers operating at 488 nm and 635 nm. The resulting data were processed using FlowJo software (TreeStar, Ashland, OR, USA). Blood lymphocytes were selected based on their SSC/FSC characteristics. After the red blood cells were removed, the remaining cells were suspended in respective antibody diluents of CD4+-PE and CD8+-FITC, and then incubated in the dark. The fluorescence-tagged lymphocytes were detected under consistent compensation settings.

### 2.11 Real-time PCR analysis

Total RNA was isolated from fresh samples employing the FastPure^®^ Cell/Tissue Total RNA isolation kit (Vazyme, Nanjing, China). A quantity of 1 μg of total RNA was converted into cDNA utilizing the EZscript Reverse Transcription Mix II (EZBioscience, Suzhou, China) following the manufacturer’s guidelines. The qRT-PCR analysis was conducted on the ABI7500 system with the Polarsignal^®^ SYBR Green qPCR mix (MIKX, Shenzhen, China). All data were normalized against β-actin expression levels. The relative expression of RNA was determined using the 2^-ΔΔCT^ method as described by Livak and Schmittgen in 2001 (20). Comprehensive details regarding the primers can be found in **Supplementary Table S1**. All primers were validated by a DL2000 DNA marker (Tsingke, DLE101, Beijing, China).

### 2.12 Statistical analysis

All data were presented as mean ± SEM. Statistically significant differences were defined as *P* < 0.05, using a one-way analysis of variance (ANOVA) with the statistical software SPSS13.0 (StatSoft, Tulsa, OK, USA).

## 3 Results and discussion

**Figure 1:**
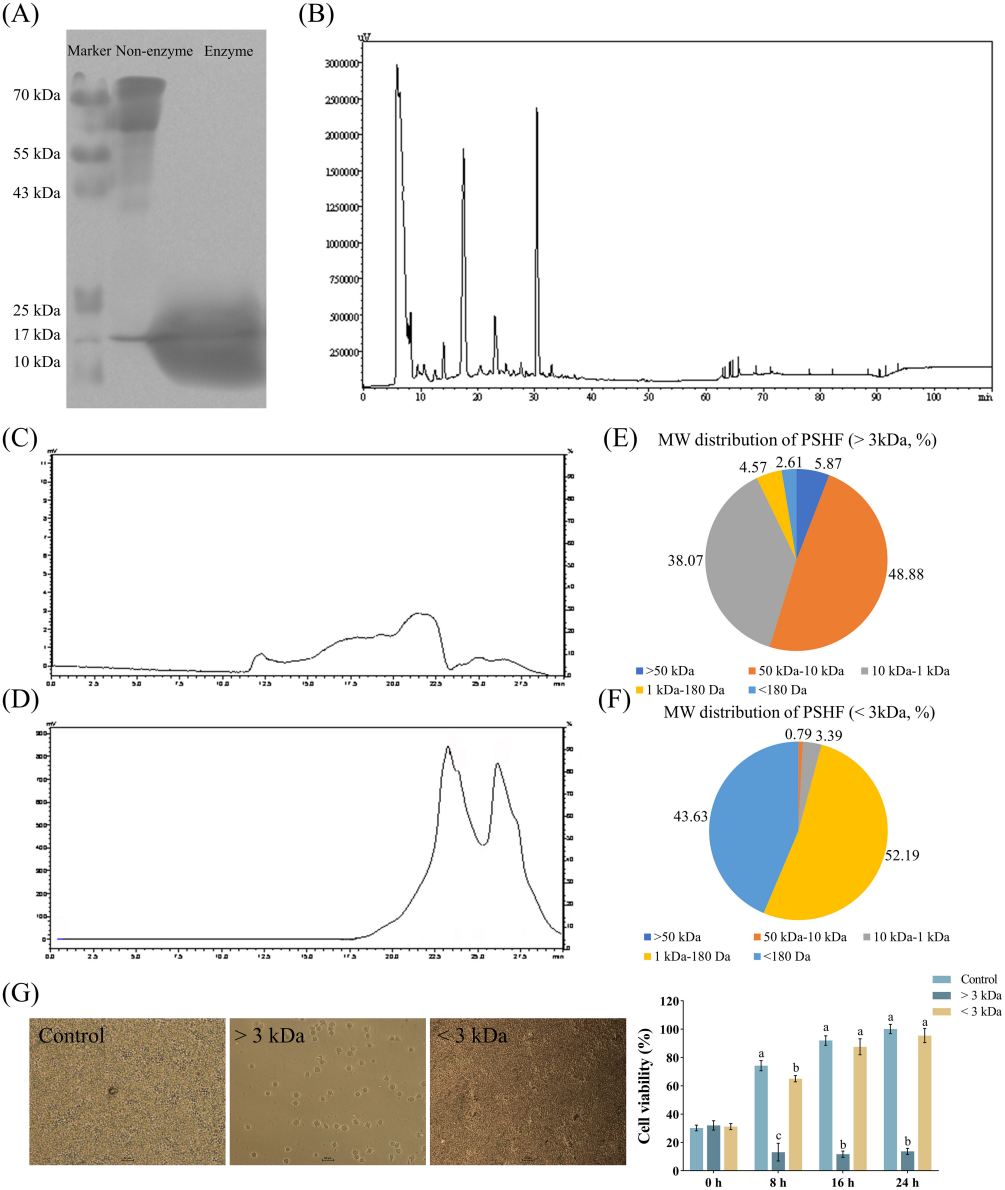
Extraction and separation of pig spleen-derived hydrolysates fractions (PSHF). **(A)** PSHF extracted from SDS-PAGE. **(B)** PSHF separated by HPLC. **(C, D)** PSHF with MW > 3 kDa at the top and MW < 3 kDa at the bottom from GPC. **(E, F)** The distribution of PSHF with varying MWs. **(G)** The morphology and viability of RAW 264.7 cells treated with PSHF between MWs > 3 kDa and < 3 kDa in Exp.1. Graph bars marked with different letters on top represent statistically significant results (*P* < 0.05) using one-way ANOVA analysis. Data were presented as mean ± SEM (4 replicates per group in three independent experiments).

There are several methods for preparing small peptides, of which the most common one is the enzymatic hydrolysis method. This method is widely recognized for its numerous advantages, including high safety, mild hydrolysis conditions, ease of process control, simple equipment requirements, and low production costs. Various factors, such as enzyme specificity, hydrolysis time, enzyme/substrate ratio, and substance concentration, significantly influence the yield, composition, and functional activity of the produced bioactive peptides. In the present study, the cleavage of peptides derived from pig spleen was clearly attributed to alkaline protease and aminopeptidase. The results of approximate MW distribution of the splenic peptide digest products (**Figure 1A**) indicated that the composition of total proteins in the supernatant exhibited the notable differences between before and after the digestion process. Protein bands in the <10 kDa region were observed following the restricted enzyme digestion treatment, whereas those in the non-digested protein were concentrated in the >50 kDa region. This finding was in line with the peptide size distribution reported for alkaline proteases in sodium casein (21) and wheat protein (22). Furthermore, the enzymatic hydrolysis of wheat germ albumin demonstrated that the digestive effect of neutral proteases was less effective than that by alkaline proteases (23). The varying enzymatic effects highlighted the necessity of selecting an appropriate type of protease for the enzymatic hydrolysis of proteins to effectively extract bioactive peptides (24). Enzymatic hydrolysis to produce bioactive peptides is often preferable to other methods due to the short reaction time, ease of scalability and predictability. Furthermore, the HPLC analysis (**Figure 1B**) revealed that the primary peak occurred within the first 30 minutes, consistent with previous findings (15). To obtain enzymatic products with varying MWs, a 3 kDa ultrafiltration membrane was employed to facilitate the separation of these products, with the molecular sizes of the isolated fractions further analyzed using GPC. As shown in **Figures 1C–F**, ultrafiltration successfully separated the enzymatic products into two distinct fractions, with the >3 kDa fraction comprising 92.82% and the <3 kDa fraction accounting for 95.82%. It is well established that most bioactive peptides exhibiting strong antitumor and antioxidant properties demonstrate lower MWs *in vitro* (25, 26). In our study, it was evident that hydrolysates with an MW of less than 3 kDa exhibited greater cell viability compared to those exceeding 3 kDa after the incubation of 8 h (**Figure 1G**). This finding suggested that the potential positive effect of the hydrolysates may be confined to bioactive components within the fractions below 3 kDa. Supporting evidence from studies on porcine plasma proteins (< 3 kDa) and royal jelly proteins (< 1 kDa) indicates that the low MW fractions of hydrolysates display the highest antioxidant activity *in vitro* (27, 28). In addition, the short peptide fraction (< 3 kDa) derived from *Schizochytrium* sp. protein hydrolysate, which was processed using alcalase and flavourzyme, exhibited superior antioxidant effects *in vivo* compared to other peptide fractions (29). This enhanced activity was attributed to the capacity of smaller peptides, which are non-toxic and highly stable, to penetrate cells or tissues and interact with target molecules (29). Furthermore, peptides with lower MWs exhibited greater structural flexibility, thereby facilitating the exposure of the active sequence and consequently enhancing their biological activity (30, 31).

**Figure 2:**
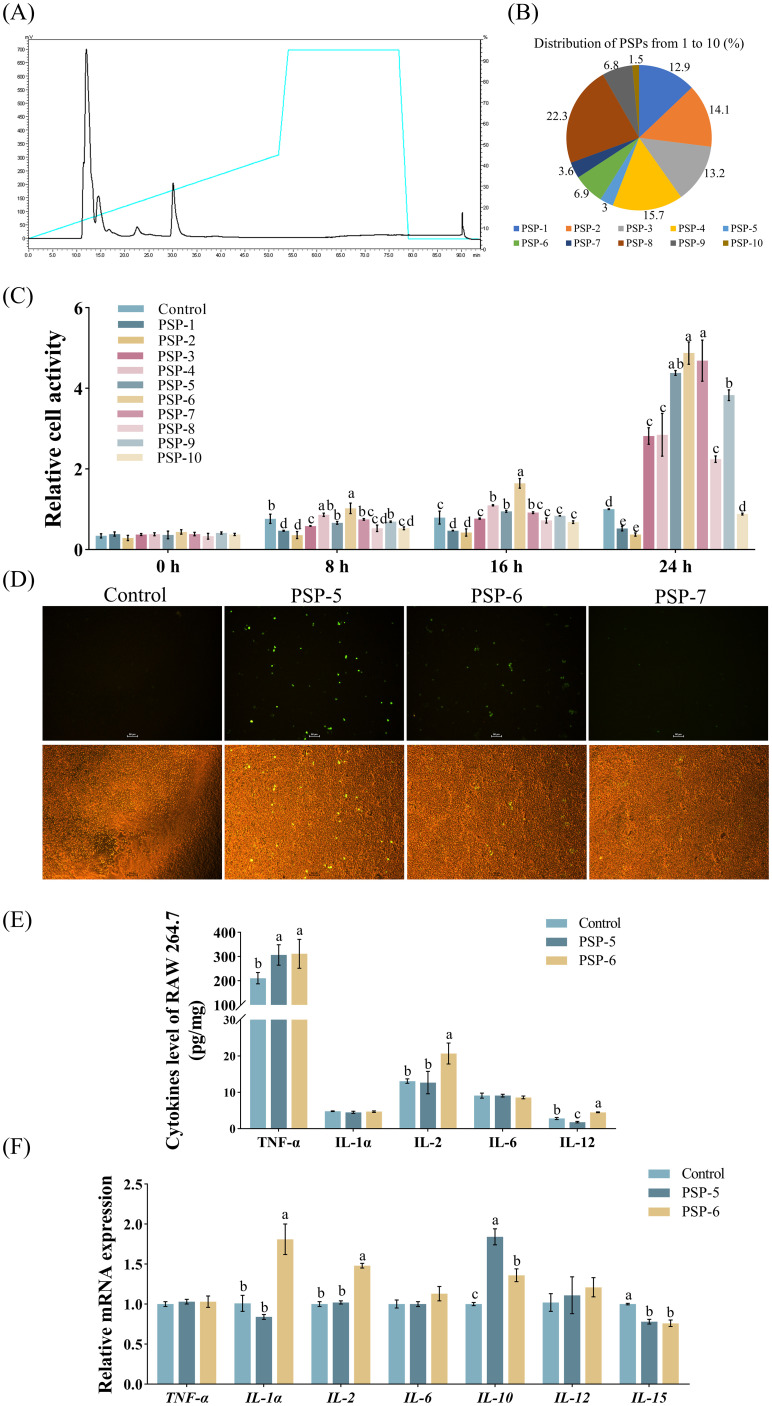
The separation and evaluation of immunomodulatory properties in pig spleen-derived peptides (PSPs). Graph bars marked with different letters on top represent statistically significant results (*P* < 0.05) using one-way ANOVA analysis. Data were presented as mean ± SEM (4 replicates per group in three independent experiments). **(A)** PSP fractions screening from HPLC with the main peaks from 1, 2, 3, 4, 5, 6, 7, 8, 9 and 10 (from PSP-1 to PSP-10). **(B)** The distribution of PSPs from 1 to 10. **(C)** Effect of PSPs from 1 to 10 on the viability of RAW264.7 cells in Exp.2 (n = 4). **(D)** Effect of PSPs from 5 to 7 on NO synthase activity. **(E-F)** Cytokine levels and gene expression in cells treated with PSP-5 and PSP-6 in Exp.2.

The immunomodulatory characteristics of small peptide fractions derived from the enzymatic hydrolysis of pig spleen were examined using RAW 264.7 macrophages. Initially, hydrolysates with a MW of less than 3 kDa were isolated via HPLC (**Figure 2A**), yielding ten distinct fractions designated as PSP-1 through PSP-10, with compositions of 12.9%, 14.1%, 13.2%, 15.7%, 3.0%, 6.9%, 3.6%, 22.3%, 6.8%, and 1.5%, respectively (**Figure 2B**). The incubations of fractions PSP-5, PSP-6, and PSP-7 exhibited more pronounced beneficial effects on cell viability compared to the other fractions (**Figure 2C**). Furthermore, *in vitro* investigations demonstrated that PSP-5 and PSP-6 displayed higher fluorescence levels of NO in RAW 264.7 macrophages than PSP-7 (**Figure 2D**). It has been noted that peptide chains can generate a variety of functional bioactive products through different proteases, which was attributed to numerous cleavage sites (32). Distinct immune responses have been reported in peptides derived from wheat germ (33) and sodium casein (34). Additionally, the incubation of PSP-5 and PSP-6 resulted in increased production of TNF-α and IL-2 (**Figure 2E**), along with an upregulation of mRNA expressions for *IL-1α*, *IL-2*, and *IL-10*, while reducing *IL-15* mRNA levels in comparison to the control group (**Figure 2F**), suggesting that the immune process could be effectively activated to fight infection. It is confirmed that the incubation of sodium caseinate and hydrolysate derived from wheat gluten proteins has been shown to promote the expression of inflammatory cytokines, thereby stimulating immune processes *in vitro* (35–37). Furthermore, the immunomodulatory effects were enhanced by PSP-5, which demonstrated lower *IL-12* production and higher *IL-10* mRNA expression compared to PSP-6 in macrophages. The ability of bioactive peptides to modulate the immune response is influenced by factors such as their amino acid composition, sequence, length, hydrolysis degree, and molecular structure.

**Figure 3:**
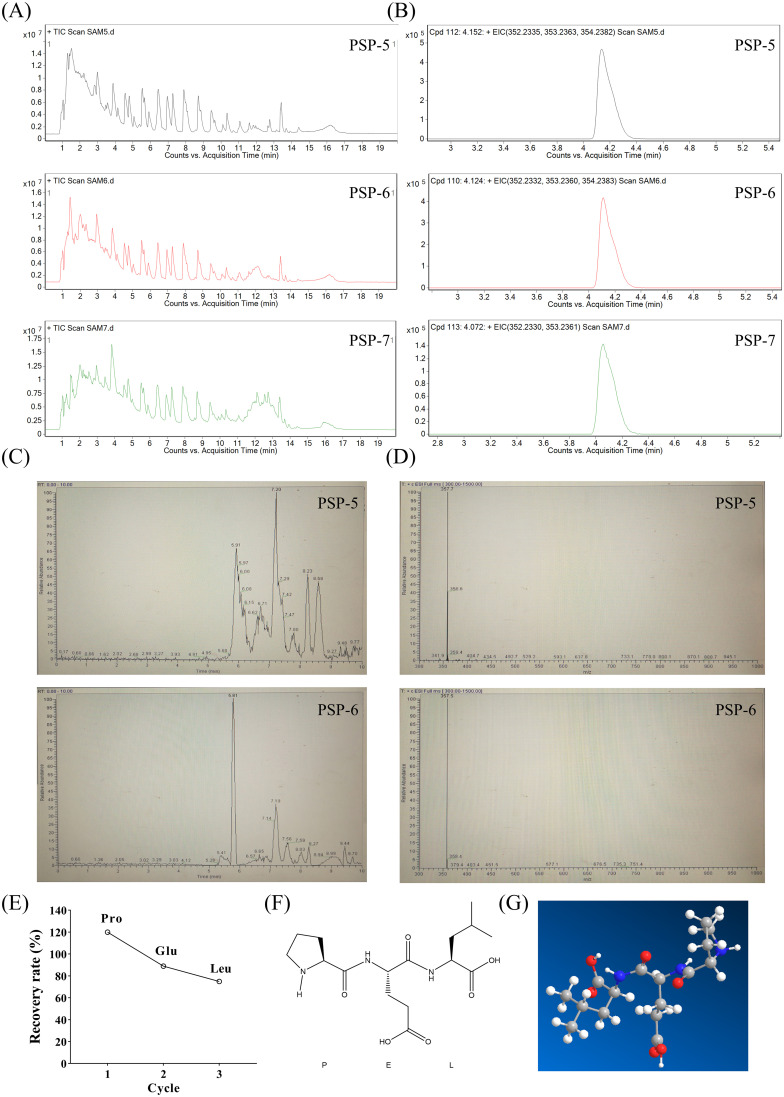
Identification of molecular mass and amino acid sequences of target fragments in PSP-5 and 6. **(A)** Total ion chromatogram of PSP-5, 6 and 7 using LC-MS. **(B)** Extracted ion chromatogram of PSP-5, 6 and 7. **(C)** Chromatograms of the different fragments from PSP-5 and PSP-6. **(D)** Primary mass spectrometry of the same fragment of PSP-5 and PSP-6. **(E)** Recovery rate of the most abundant amino acids of Pro, Glu and Leu. **(F-G)** Mapping of small peptide structures and fitting prediction of 3D structures using PPSQ.

While PSP-5, PSP-6, and PSP-7 demonstrated macrophage activity, only PSP-5 and PSP-6 effectively induced the production of NO. Consequently, a comparative analysis of their compositional differences was performed using Q-TOF-MS (**Figure 3A**), which identified a fraction enriched in PSP-5 and PSP-6 but less abundant in PSP-7 (**Figure 3B**). After confirmation of the target small peptide, further separation via LC-MS (**Figures 3C, D**) and amino acid sequencing through PPSQ were conducted, revealing the amino acid sequence to be Pro-Glu-Leu (**Figure 3E**). The chemical structure of the small peptide (**Figure 3F**) and its predicted three-dimensional structure (**Figure 3G**) are also illustrated. The biological activities of peptides are closely associated with their secondary structures (38). It has been shown that peptide sequences containing the charged heterocyclic amino acid Pro can enhance the antitumor effects of specific peptides (39). Additionally, Vogel et al. (40) indicated that the immunomodulatory and anti-inflammatory properties of lactoferricin peptides are primarily linked to a positively charged region of the peptide, which interacts with and activates immune cell chemokine receptors. The presence of Pro and Glu in numerous immunomodulatory peptides (11) implies that this small peptide may play a critical role in the immunomodulatory effects observed in PSP-5 and PSP-6.

**Figure 4:**
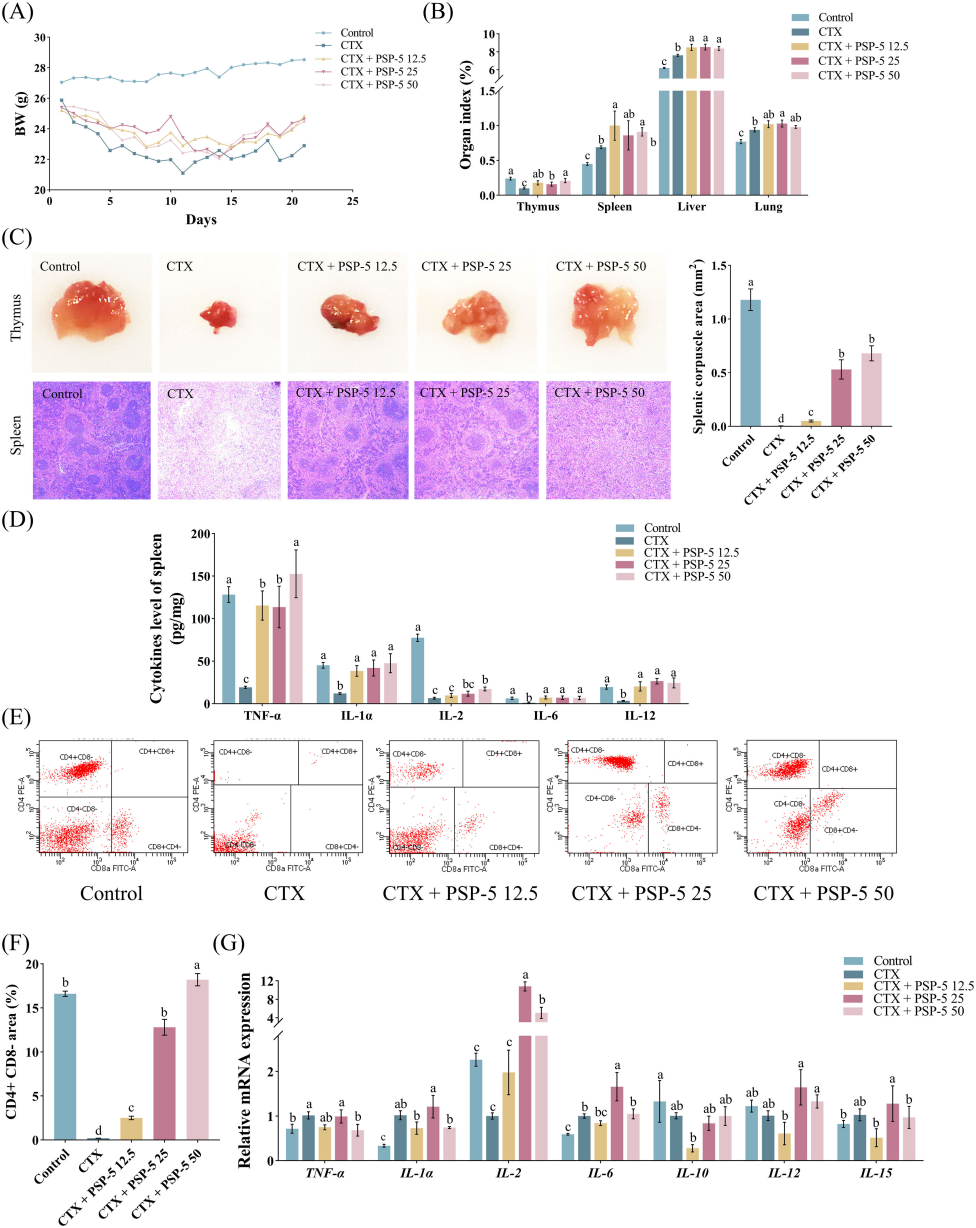
PSP-5 mitigates CTX-induced immunosuppression in spleen of mice in Exp.3. Graph bars marked with different letters on top represent statistically significant results (*P* < 0.05) using one-way ANOVA analysis. Data were presented as mean ± SEM Data were presented as mean ± SEM (4 replicates per group). **(A)** Body weight. **(B)** Immune organ index. **(C)** Morphological evaluation of thymus and spleen. **(D)** Levels of inflammatory cytokines in spleen. **(E-F)** Subpopulations of T-lymphocyte in blood. **(G)** Gene mRNA expressions related to inflammatory cytokines in spleen.

**Figure 5:**
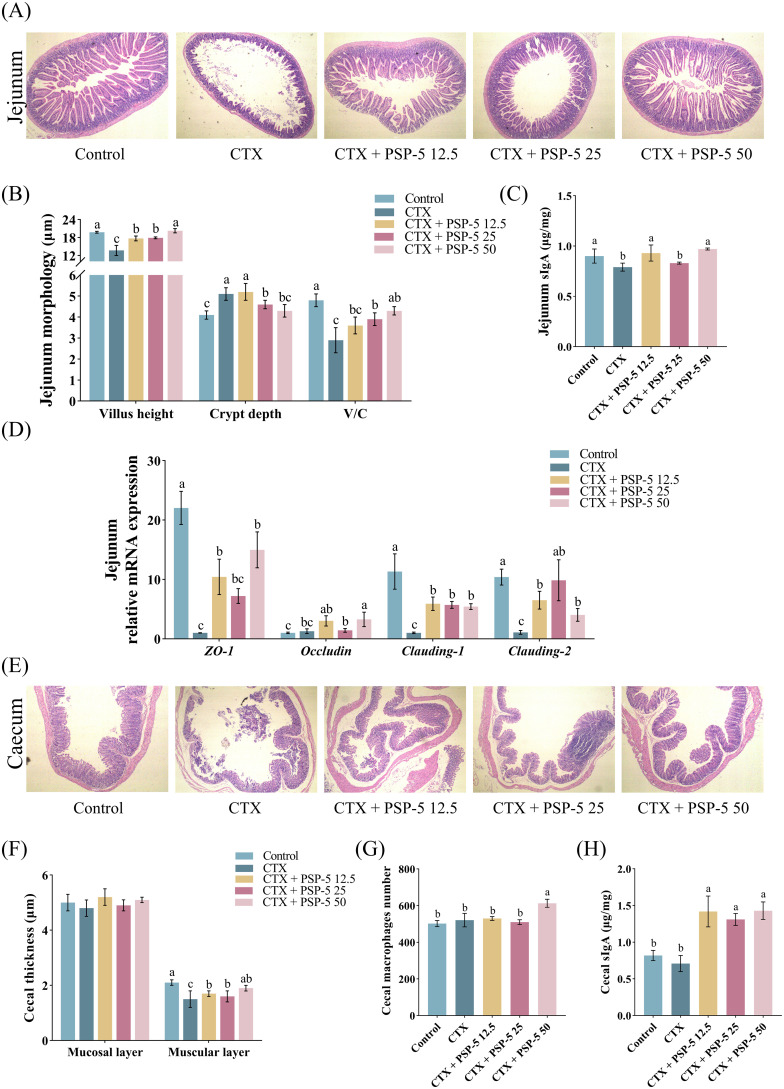
PSP-5 mitigates CFX-induced the damage of barrier function in intestine of mice. Graph bars marked with different letters on top represent statistically significant results (*P* < 0.05) using one-way ANOVA analysis. Data were presented as mean ± SEM (4 replicates per group). **(A)** Representative pictures of HE staining of jejunum. **(B)** Villus height, crypt depth, and villus-crypt ratio in jejunum. **(C)** The level of sIgA in jejunum. **(D)** Gene mRNA expressions of tight junction proteins in jejunum. **(E)** Representative pictures of HE staining of cecum. **(F)** Thickness of mucosal layer and muscular layer in cecum. **(G)** The number of cecal macrophages. **(H)** The level of sIgA in cecum.

To investigate the structure-activity relationship, we examined the bioactivity potential of the biological agent PSP-5, derived from pig spleen, *in vivo* using a mouse model. Cyclophosphamide (CTX), a potent immunosuppressive compound, is known to suppress both humoral and cell-mediated immune responses (41). Previous studies have indicated (42) that administering a CTX dose of 100 mg/kg resulted in reduced body growth and increased relative weights of the spleen, liver, and lungs in mice compared to a control group treated with saline. Conversely, administering escalating doses of PSP-5, ranging from 5 to 50 mg/kg orally, resulted in increased body weight (**Figure 4A**) and promoted the development of immune organs (**Figure 4B**) in mice subjected to CTX-induced stress. Furthermore, the HE staining results revealed that PSP-5 exerted protective effects against CTX-induced immunosuppression in a dose-dependent manner in repairing structural damage, as characterized by the decreased vacuolation, inflammation, and necrosis observed in the spleen following CTX administration (**Figure 4C**). Administering 25-50 mg PSP-5/kg body weight has been shown to effectively reverse the reductions in the production of TNF-α, IL-1α, IL-6, and IL-12 induced by CTX in the spleen (**Figure 4D**). Additionally, PSP-5 restores both the area and density of CD4+ and CD8- T cells in the bloodstream (**Figures 4E, F**) to the normal levels of mice given saline orally. The enhancement of immune capability by PSP-5 *in vivo* is further supported by *in vitro* experiments. The immunomodulatory effects of PSP-5 on CTX-induced immunosuppression might be associated with the activation of macrophages and T cells, which are well-established targets for immunomodulatory peptides (38). We propose that PSP-5 can enhance immune system efficacy by directly interacting with receptors on the surfaces of immune cells, thereby promoting the production of downstream inflammatory mediators. However, no corresponding changes were observed in the mRNA expression levels of relevant cytokine genes (**Figure 4G**). Numerous studies have indicated that smaller peptides, such as di- and tripeptides, are more readily absorbed in the intestine, thereby facilitating their immunomodulatory effects against immune suppression within tissues. In the present study, the morphological characteristics of the jejunum were compromised in the presence of CTX, as indicated by a reduction in villus height (**Figures 5A, B**) and decreased sIgA secretion (**Figure 5C**), along with an increase in crypt depth, which normalized upon administration of PSP-5 orally. Notably, the intervention with PSP-5 led to a substantial increase in the expression of mRNA for *Claudin*-1, *Claudin-2*, and *ZO-1*, which were associated with tight junction function in the jejunum of CTX-stimulated mice (**Figure 5D**). Moreover, oral administration of PSP-5 effectively countered the reduction in wall thickness (**Figures 5E, F**). In addition, the number of macrophages (**Figure 5G**) and the level of sIgA (**Figure 5H**) in the caecum following CTX treatment. This suggests that the peptide may withstand potential endogenous degradation, be assimilated into the serum via intestinal cells, and effectively reach and engage with target tissues for functional activity. These protective benefits resulting from small peptides were consistent with findings in mice (43), rats (44), and chickens (45). Given that pig spleen models exhibit significant anatomical and physiological similarities to the immune system in humans, it was speculated that there was potential health benefits of PSP-5 peptide and the applications in various human nutrition products.

In summary, we firstly reported that a novel of the pig spleen-derived bioactive immunopeptides (PSP-5 and 6) by enzymatic hydrolysates, which was mainly composed of Pro-Glu-Leu, exhibited a critical role of the immunomodulatory properties in the increases of cell viability and the activation of anti-inflammatory process *in vitro*. The oral administration of 20-50 mg PSP-5/kg BW effectively protect against CTX-induced immunosuppression as characterized by the increase of immunological activity in spleen and barrier function of intestine *in vivo*. Further studies are essential to elucidate the molecular mechanisms underlying these bioactive peptides to enhance immune function and support intestinal health.

## Data Availability

The data are available from the corresponding author on reasonable request.
