# Supplementary material for: Preparation and characterization of immunopeptides isolated from pig spleen and evaluation of their immunomodulatory properties in vitro and in vivo
Source: Front Immunol. 2025 Mar 18;16:1544299. doi: 10.3389/fimmu.2025.1544299 (PMC11959066; doi:10.3389/fimmu.2025.1544299)
Supplement: Supplementary file 1 [file Table1.docx]

**Table S1**. The information of primers for target genes

| Genes | Primer sequence 5’-3’ | Product size (bp) |
| --- | --- | --- |
| *TNF-α* | \| F: CAGGCGGTGCCTATGTCTC \| \| --- \| \| R: CGATCACCCCGAAGTTCAGTAG \| | 89 |
| *IL-1α* | \| F: CGAAGACTACAGTTCTGCCATT \| \| --- \| \| R: GACGTTTCAGAGGTTCTCAGAG \| | 126 |
| *IL-2* | \| F: TGAGCAGGATGGAGAATTACAGG \| \| --- \| \| R: GTCCAAGTTCATCTTCTAGGCAC \| | 120 |
| *IL-6* | \| F: TCTTGGGACTGATGCTGGTGA \| \| --- \| \| R: GGACTCTGGCTTTGTCTTTCTTGT \| | 384 |
| *IL-10* | \| F: AGCCTTATCGGAAATGATCCAGT \| \| --- \| \| R: GGCCTTGTAGACACCTTGGT \| | 229 |
| *IL-12* | \| F: AGACATCACACGGGACCAAAC \| \| --- \| \| R: CCAGGCAACTCTCGTTCTTGT \| | 77 |
| *IL-15* | \| F: CATCCATCTCGTGCTACTTGTG \| \| --- \| \| R: GCCTCTGTTTTAGGGAGACCT \| | 112 |
| *ZO-1* | \| F: CAACCAGATGTGGATTTACCC \| \| --- \| \| R: GATTCTACAATGCGGCGA \| | 359 |
| *Occludin* | \| F: TTGAAAGTCCACCTCCTTACAGA \| \| --- \| \| R: CCGGATAAAAAGAGTACGCTGG \| | 129 |
| *Claudin-1* | \| F: GGGGACAACATCGTGACCG \| \| --- \| \| R: AGGAGTCGAAGACTTTGCACT \| | 100 |
| *Claudin-2* | \| F: TCCCTGGTGGTTCAGTAAGT \| \| --- \| \| R: CAGAACAAGTAAGGGAGGACA \| | 169 |
| *β-actin* | \| F: GTGACGTTGACATCCGTAAAGA \| \| --- \| \| R: GCCGGACTCATCGTACTCC \| | 245 |

F：Forward; R: Reverse
